# Enhanced recovery after surgery or fast-track surgery and the perioperative period of acute gastrointestinal perforation: a systematic review and meta-analysis

**DOI:** 10.3389/fsurg.2025.1529279

**Published:** 2025-04-11

**Authors:** Chuan Wang, Wenna Qiu, Hailong Qu, Pengfei Li, Wei Xu, Yanwei Fang

**Affiliations:** ^1^ Department of Emergency Surgery, The Second Hospital of Hebei Medical University, Shijiazhuang, Hebei, China; ^2^ Department of Neonatology, Hebei Children’s Hospital, Shijiazhuang, Hebei, China; ^3^ Department of Interventional Catheter, Yi County Hospital of Traditional Chinese Medicine, Baoding, Hebei, China; ^4^ Department of Spinal Surgery, People’s Hospital of Hengshui, Hengshui, Heibei, China

**Keywords:** perioperative period, enhanced recovery after surgery, fast-track surgery, acute gastrointestinal perforation, meta-analysis, systematic review

## Abstract

**Background:**

Reports of an association between enhanced recovery after surgery (ERAS) or fast-track surgery (FTS) and the perioperative period of acute gastrointestinal perforation are inconsistent. Therefore, we systematically evaluate the safety and efficacy of ERAS or FTS in the perioperative of acute gastrointestinal perforation.

**Methods:**

Randomized controlled trial (RCT) or controlled clinical trial (CCT) on the application of ERAS/FTS in the perioperative management of acute gastrointestinal perforation was conducted by PubMed, Medline, Web of Science, Ovid, Elsevier ScienceDirect, Cochrane Library, Embase, China National Knowledge Infrastructure (CNKI), Chinese Biomedical Database (CBM), Wanfang Data, and WHIP. The methodology quality and data extraction were evaluated by two researchers, and meta-analysis was performed by Stata 11 software.

**Results:**

A total of 20 RCTs and 7 CCTs were included in the study, involving 1,864 patients—917 in the ERAS/FTS group and 947 in the control group. The results of the meta-analysis showed that the stress response CRP and complication rate of the ERAS/FTS group were significantly lower than those of the traditional treatment group, the time of first out-of-bed activity and the time of postoperative first exhaust and eating were advanced, and the cost and the length of hospital stay were decreased (*p* < 0.05). Egger's test showed no publication bias (*p* > 0.1). However, only two and three studies mentioned operative time and pain management, respectively, so the meta-analysis could not be performed.

**Conclusion:**

The application of ERAS/FTS in perioperative management of acute gastrointestinal perforation is safe and effective.

## Introduction

Acute digestive tract perforation is a common surgical emergency, most often caused by an ulcer in the upper digestive tract. This condition has a rapid onset, is critical, and progresses quickly. When perforation occurs, gastrointestinal contents leak into the abdominal cavity, causing serious significant contamination. This can easily lead to internal environment disorders and severe stress reactions during the perioperative period. In severe cases, it can result in life-threatening septic shock, often necessitating emergency surgery. Complications have not yet reached a level of public satisfaction (1). For example, early feeding may increase the incidence of anastomotic dehiscence, particularly in critical, emergency, elderly, and malnourished patients (2). This has long been a concern for many surgeons.

Enhanced recovery after surgery (ERAS) is a comprehensive multidisciplinary approach to improve a series of routine diagnostic and therapeutic measures; to reduce the operative stress, risk, and complications; and finally to accelerate the postoperative recovery, improve the quality of rehabilitation, shorten the hospital stay (3, 4). ERAS has been gradually extended from its initial application mainly in colorectal surgery to almost all surgical fields (5). But up to now, ERAS is still mainly used in elective surgery and is relatively late in emergency surgery. Reviewing the published ERAS studies reveals that most of them focus on elective surgery in young patients without severe comorbidities. However, there are limited applications and research in critical, emergency, elderly, and malnourished patients. These patients often face more complex surgical scenarios and experience more severe stress consequences (2). Additionally, few studies exist on the application of ERAS during the perioperative period for gastrointestinal perforation, unlike colorectal surgery, which has established guidelines and expert consensus (6, 7). This lack of research hinders the development of ERAS and its acceptance among medical professionals. Therefore, optimizing perioperative treatment measures to minimize stress damage is crucial and necessary. This study was designed to systematically evaluate the safety and efficacy of the ERAS in the perioperative of acute gastrointestinal perforation by searching the literature of randomized controlled trials (RCTs) or controlled clinical trials (CCTs), to provide reliable evidence-based medicine for the clinical basis.

## Methods

### Retrieve policy

The ERAS/fast-track surgery (FTS) literature was searched by PubMed, Medline, Web of Science, Ovid, Elsevier ScienceDirect, Cochrane Library, Embase, China National Knowledge Infrastructure (CNKI), Chinese Biomedical Database (CBM), Wanfang Data, and WHIP, and languages include Chinese and English. The keywords of the database were “enhanced recovery after surgery, ERAS, fast track surgery, accelerated rehabilitation surgery, rapid rehabilitation surgery and acute gastrointestinal perforation, perforation of the digestive tract, traditional care, standard care.” An expanded search was conducted for references, relevant reviews, or case reports.

### Inclusion criteria of literature

The inclusion criteria were as follows: (1) the type of study was randomized controlled trial (RCT) or controlled clinical trial (CCT); (2) the subjects were patients who underwent acute gastrointestinal perforation surgery; (3) the patients in the ERAS/FTS group were treated with enhanced recovery after surgery, while the patients in the control group were treated with traditional perioperative management; and (4) the study reported at least one outcome measure, such as postoperative stress and inflammation (PCT/CRP/PA), operative time, intraoperative blood loss, exhaust time, first enteral nutrition time, first out-of-bed activity time, anesthesia/pain management, nursing management, hospital stay, postoperative complication rate, and hospital cost.

### Exclusion criteria of literature

The exclusion criteria were as follows: (1) the sample size of a single study was <10 cases; (2) review, case report, and single cohort studies; (3) republished literature from the same research center or the same author; (4) no relevant and available data in the literature; and (5) repeated or obviously incorrect data.

### Literature quality evaluation

Two investigators independently evaluated the included literature according to the method introduced by Athanasiou et al. (8), and in the event of disagreement, it was decided by the participation of a third investigator in the discussion. The evaluation included three aspects and nine indicators: (1) study design (RCT, inclusion criteria, and sample size); (2) comparability (age and sex, number of ERAS measures, and follow-up time); and (3) result evaluation (operation and postoperative condition, complication, and mortality). The data of the above indexes were extracted, and one item was recorded as a “*” sign. The quality was considered good if the results were more than six “*.”

### Data extraction

The full text of the included literature was read by two researchers, and relevant data were extracted according to a predesigned data extraction table. The main findings were as follows: (1) general data (title, first author, date of publication, and literature source, as shown in Table 1); (2) study characteristics (study design, sample size, age, sex, and intervention measures, as shown in Table 1); and (3) outcome measures (postoperative stress inflammation index (PCT/CRP/PA), operative time, intraoperative bleeding, exhaust time, first enteral nutrition time, first out-of-bed activity time, anesthesia/pain management, nursing management, hospitalization time, postoperative complication rate and hospitalization expense, as shown in Figures 2–8). If the literature continuity data were presented as median vs. interquartile range, these were converted to the x ± s with reference to the study by Hozo et al. (9).

**Table 1:** Characteristics of the studies included.

Study	Year	Study design	Number		Age		Male/female		ERAS interventions	Quality of literature (scores)
			ERAS/FTS	CC	ERAS/FTS	CC	ERAS/FTS	CC		
Lin et al. (2)	2023	RCT	35	35	43.71 ± 5.08	43.68 ± 5.1	18/17	20/15	1, 2, 3, 5, 7, 9, 10, 11, 12, 17, 18, 19	7*
Li et al. (10)	2022	RCT	34	34	43.14 ± 18.08	42.35 ± 17.95	14/20	15/19	1, 5,6,7,9,10,11,12,17,18,19	7*
Liu et al. (11)	2017	RCT	16	16	42.3 ± 7.8	42.3 ± 8.7			1, 2, 5, 8, 9, 11, 12, 17, 18	6*
Jia et al. (12)	2020	RCT	30	30					2, 5, 6, 8, 9, 10, 11, 12, 17, 18	7*
Huang et al. (13)	2018	RCT	24	23	39.4 ± 5.1	40.8 ± 6.3	15/9.0	13/10.0	5, 6, 7, 8, 9, 10, 11, 12, 17, 18	7*
Wu et al. (14)	2021	RCT	30	30	66.87 ± 12.83	68.57 ± 5.93	18/12.	19/11.	1, 3, 4, 5, 6, 7, 9, 10, 11, 12, 17, 18	7*
Luo et al. (15)	2018	RCT	23	24	69.0 ± 5.0	67.0 ± 6.0	15/8.	18/6.	1, 2, 5, 6, 7, 8, 9, 10, 11, 12, 17, 18	7*
LI et al. (16)	2018	RCT	40	40	69.0 ± 4.0	68.0 ± 5.0	23/17	21/19	1, 2, 5, 6, 7, 8, 9, 10, 11, 12, 17, 18	7*
Chen et al. (17)	2019	RCT	33	33	45.79 ± 3.11	46.28 ± 3.27	18/15	19/14	1, 2, 5, 6, 7, 8, 9, 10, 11, 12, 17, 18	7*
Wang et al. (18)	2020	RCT	41	41	48.78 ± 9.76	48.54 ± 9.84	23/18	24/17	1, 2, 5, 6, 7, 8, 9, 10, 11, 12, 17, 18	7*
Liu et al. (19)	2019	RCT	40	40	54.8 ± 11.7	52.1 ± 13.0	31/9	33/7.0	1, 2, 5, 6, 7, 9, 10, 11, 17, 18	8*
ZHANG et al. (20)	2020	RCT	41	41	48.25 ± 4.62	48.46 ± 4.28	25/16	26/15	2, 5, 6, 7, 8, 9, 10, 11, 12, 17, 18	7*
Chen et al. (21)	2015	RCT	34	30					2, 5, 6, 7, 8, 9, 10, 11, 12, 17, 18	6*
Yuan et al. (22)	2014	RCT	30	30	44.0 ± 2.0	45.0 ± 1.0	28/2.0	29/1.0	1, 3, 5, 6, 7, 8, 9, 10, 11, 12, 17, 18	7*
Luo et al. (23)	2014	RCT	36	36	42.1 ± 1.0	42.8 ± 2.1	20/16	22/14	2, 5, 8, 9, 10, 11, 12, 17, 18	5*
Liu et al. (24)	2019	RCT	70	70	48.22 ± 8.21	48.42 ± 9.12	38/32	36/34	1, 6, 9, 10, 11, 12, 18	6*
Tan et al. (25)	2013	RCT	27	20	31.3 ± 4.7	28.5 ± 6.3	19/8.0	14/6.0	2, 5, 6, 7, 8, 9, 10, 11, 12, 17, 18	8*
LIU et al. (26)	2013	RCT	30	30	42.1 ± 1.0	42.8 ± 2.1	30/0	30/0	5, 6, 7, 8, 9, 12, 13, 17, 18	7*
Cao et al. (27)	2016	RCT	39	39					2, 5, 6, 7, 8, 9, 10, 11, 12, 17, 18	7*
Khripun et al. (28)	2020	RCT	51	87					2, 5, 6, 7, 8, 9, 10, 11, 12, 17, 18	7*
Yu et al. (29)	2013	CCT	29	32	42.6 ± 11.1	41.8 ± 11.4			1, 2, 5, 8, 9, 11, 12, 17, 18	6*
ZHANG et al. (30)	2020	CCT	35	35	72.45 ± 12.58	71.48 ± 11.79	18/17	20/15	1, 2, 5, 6, 7, 8, 9, 10, 11, 12, 17, 18, 19	6*
QIAN et al. (31)	2018	CCT	30	30					2, 5, 6, 7, 8, 9, 10, 11, 12, 17, 18	5*
XIE et al. (32)	2012	CCT	32	30					2, 5, 6, 7, 8, 9, 10, 11, 12, 17, 18	6*
Wang et al. (33)	2014	CCT	25	25	42.1 ± 0	42.1 ± 0	25/0	25/0	1, 2, 3, 8, 9, 10, 11, 12, 17, 18	6*
SHI et al. (34)	2015	CCT	38	38	43.27 ± 11.04	45.63 ± 11.43	21/17	20/18	2, 5, 6, 8, 9, 10, 11, 12, 13, 17, 18	6*
XIE et al. (35)	2015	CCT	24	28	66.3 ± 4.2	63.2 ± 3.8	13/11.0	15/13	5, 6, 16, 17, 18	6*

ERAS interventions: 1. ERAS concept mission. 2. Preoperative sugar load. 3. Prophylactic use of antibiotics. 4. Prevention of stress mucosal lesions. 5. Early removal of gastric tube. 6. Early removal of the ureter after surgery. 7. Early removal of the drainage tube in the surgical area. 8. Anesthesia management: mid-thoracic EPIDURAL + general anesthesia (short half-life). 9. Surgical approach: laparoscopic surgery. 10. Fluid management: individualized goal-directed restrictive fluid therapy (GDFT). 11. Keep warm during surgery. 12. Pain Management: postoperative preventive, timely, and multimodal analgesia. 13. Drugs regulate inflammation. 14. Prophylactic antithromboembolism. 15. Prevention of nausea and vomiting. 16. Prevention of bowel paralysis and promotion of gastrointestinal peristalsis. 17. Get out of bed early after surgery. 18. Early postoperative water intake, gastric tube removed the day of fluid food, and gradually transition to a normal diet. 19. Personalized care.

The literature quality evaluation included 3 aspects and 9 indicators: (1) Study Design: RCT, Inclusion criteria and sample size; (2) comparability: age and sex, number of ERAS measures and follow-up time; (3) result evaluation: operation and postoperative condition, complication and mortality. The data of the above indexes were extracted, and 1 item was recorded as a “*” sign.

### Statistical analysis

Stata 11 software was used for meta-analysis. Odds ratio (OR) was used as the combined statistic for the counting data, and weighted mean difference (WMD) was used as the combined statistic for the same index, such as the results obtained with the same measuring tools. If results were obtained using different measurement tools, standard mean difference (SMD) was used as pooled statistics; 95% confidence intervals (CI) were calculated for all statistics. The heterogeneity of each study was analyzed by chi-square test, and the homogeneity studies (*p* > 0.05, *I*^2^ < 50%) were analyzed by fixed effect model. Heterogeneity studies (*p* < 0.05, *I*^2^ > 50%) were meta-analyzed by a random-effects model. Funnel plot analysis and Begg’s or Egger’s method were used to test publication bias. *p* < 0.05 indicates that the difference is statistically significant.

## Result

### Results of literature inclusion

**Figure 1:**
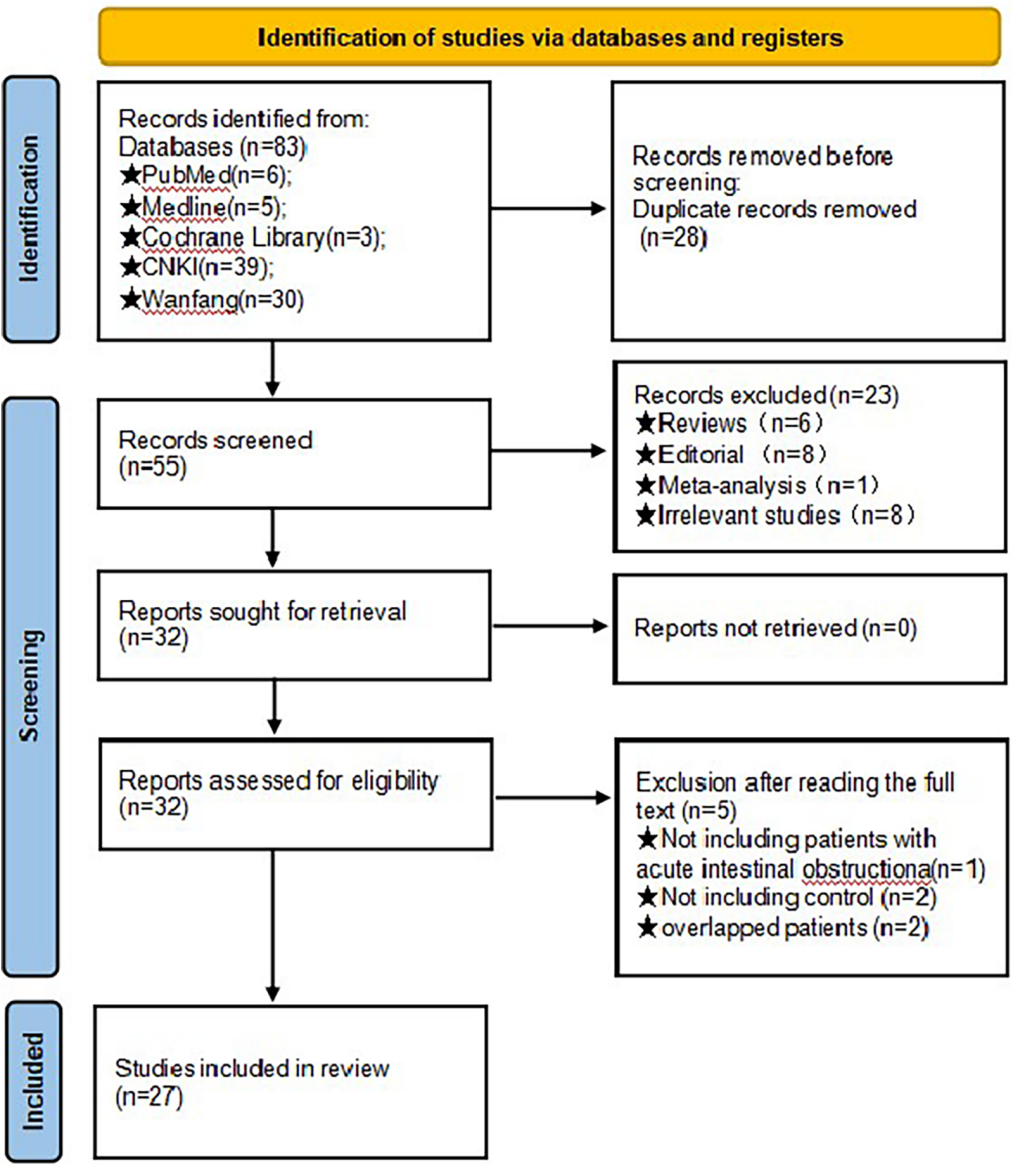
Flowchart of PRISMA search. Initially, a total of 83 records were obtained through the database search. Subsequently, 28 duplicate entries were identified and eliminated. Upon scrutinizing the titles and abstracts, 23 studies were excluded due to their lack of alignment with the meta-analysis objectives. Further examination of the full texts of the remaining 32 studies resulted in the exclusion of an additional 5 studies, with the specific rationales for exclusion outlined in figure. Ultimately, 27 studies were chosen for inclusion in the final meta-analysis.

According to the search strategy, 27 articles were finally included (2, 10–35), as shown in Figure 1: 20 RCT studies, 7 CCT studies, 1 English article, and 26 Chinese articles, involving 1,864 patients in total. There were 917 cases in the test group and 947 cases in the control group. The general data included in the literature are shown in Table 1. According to Athanasiou et al. (8), 25 articles were high quality, and the rest were low quality.

### Stress responses

**Figure 2:**
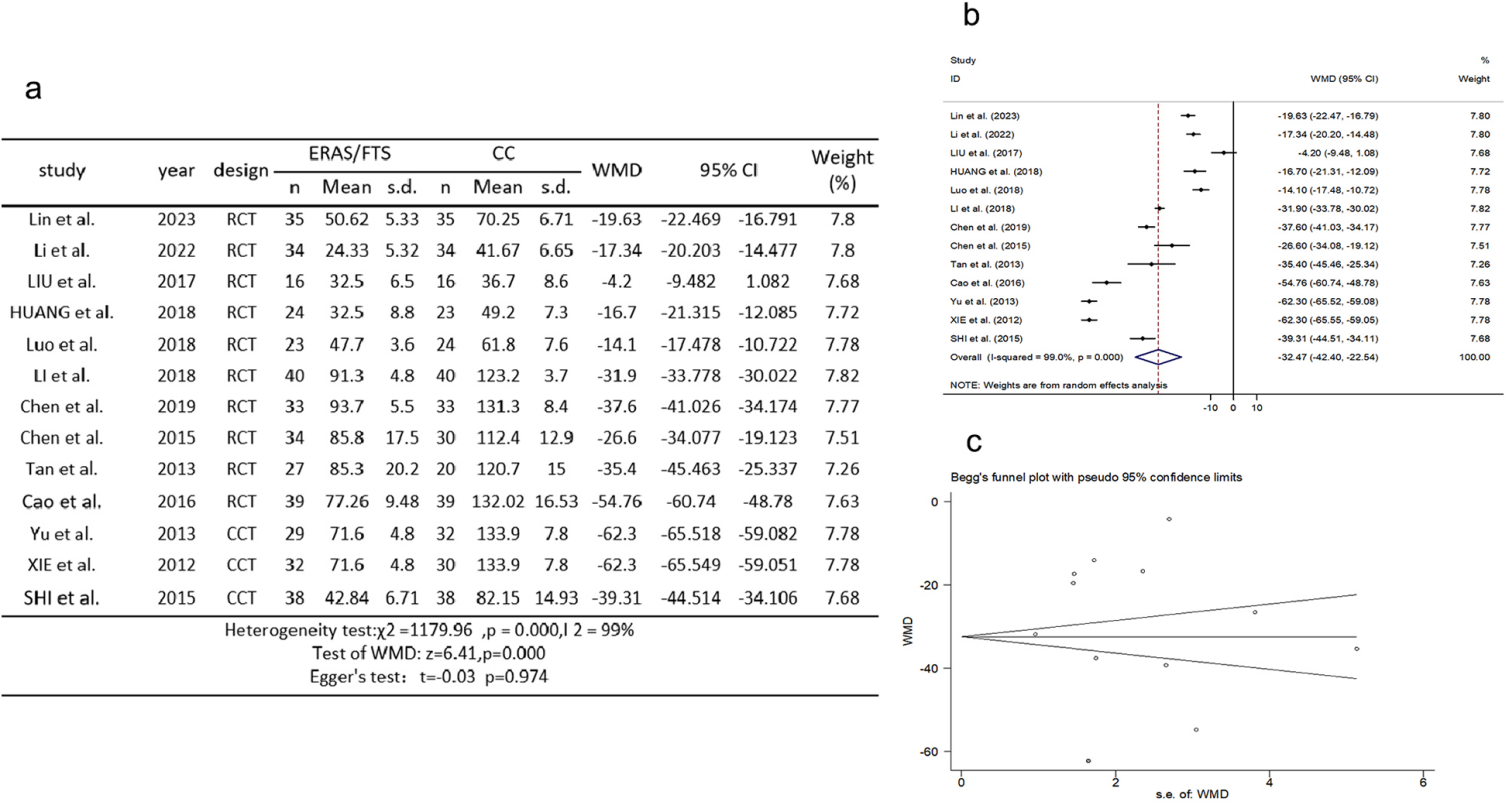
Association between ERAS or FTS and stress responses in the perioperative period of acute gastrointestinal perforation. **(a)** Results of the meta-analysis of the association between ERAS or FTS and stress responses; **(b)** odds ratio in positive for ERAS or FTS; **(c)** Egger's funnel plot of studies investigating ERAS or FTS as a risk factor.

Thirteen studies (2, 10, 11, 15–17, 21, 25, 27, 29, 32, 34) reported a comparison of stress responses to high-sensitivity C-reactive protein (hs-CRP). There was heterogeneity among the studies (*p* = 0.000, *I*^2^ = 99%). A random-effects model was used for meta-analysis. The results showed a significant reduction in stress response in the ERAS group compared with the control group (WMD = −32.469, 95% CI: −42.401 to −22.537, *p* = 0.000); Egger's test showed no publication bias (*t* = −0.03, *p* = 0.974), as shown in Figure 2.

### Time of first exhaust after operation

**Figure 3:**
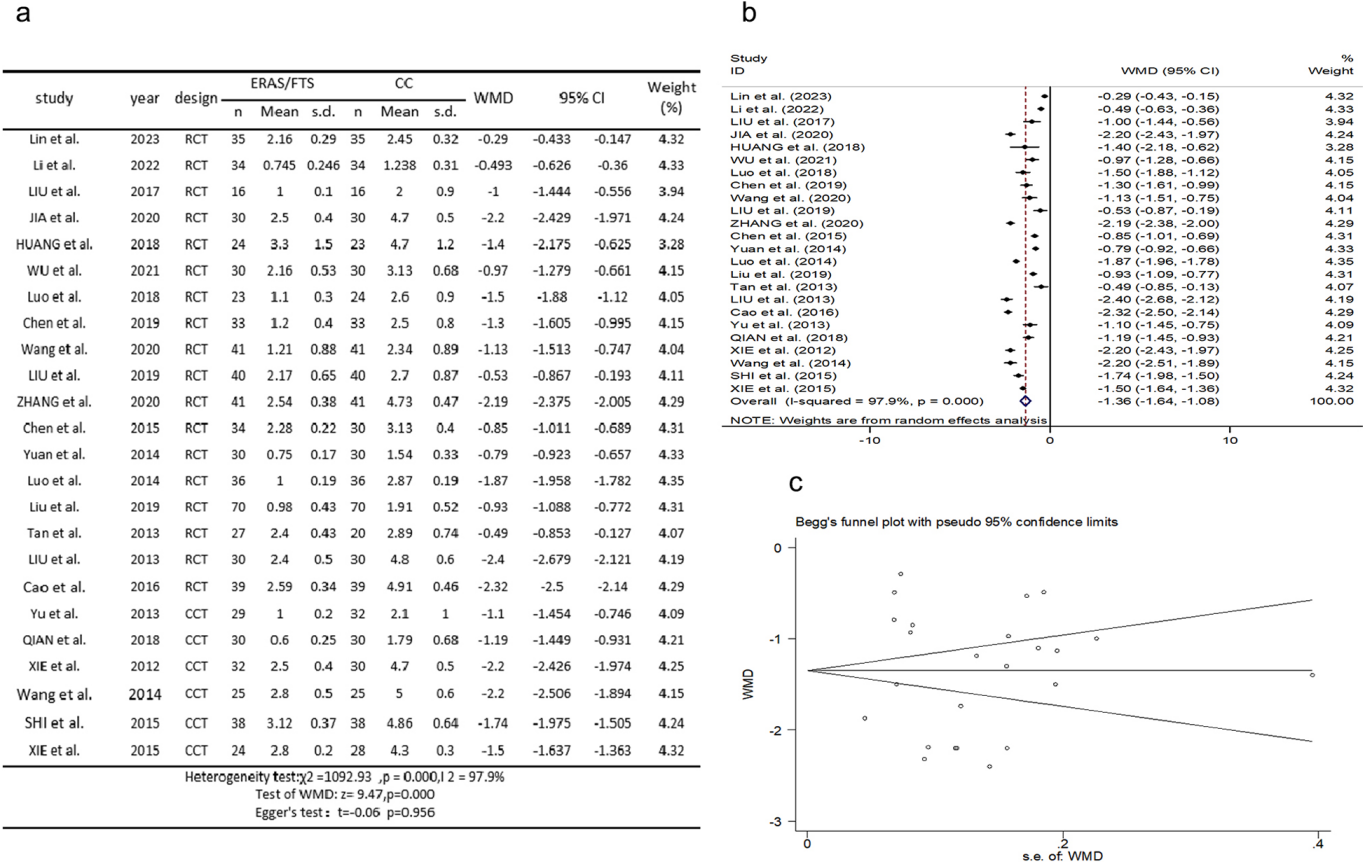
Association between ERAS or FTS and time of first exhaust after operation in the perioperative period of acute gastrointestinal perforation. **(a)** Results of the meta-analysis of the association between ERAS or FTS and time of first exhaust after operation; **(b)** odds ratio in positive for ERAS or FTS; **(c)** Egger's funnel plot of studies investigating ERAS or FTS as a risk factor.

Twenty-four studies (2, 10–15, 17–27, 29, 31–35) reported a comparison of the time to first postoperative exhaust. There was heterogeneity among the studies (*p* = 0.000, *I*^2^ = 97.9%). A random-effects model was used for meta-analysis. The results showed that the postoperative first exhaust time was earlier in the ERAS group compared with the control group (WMD = −1.360, 95% CI: −1.641 to −1.078, *p* = 0.000); Egger's test showed no publication bias (*t* = −0.06, *p* = 0.956), as shown in Figure 3.

### Time of first enteral nutrition after operation

**Figure 4:**
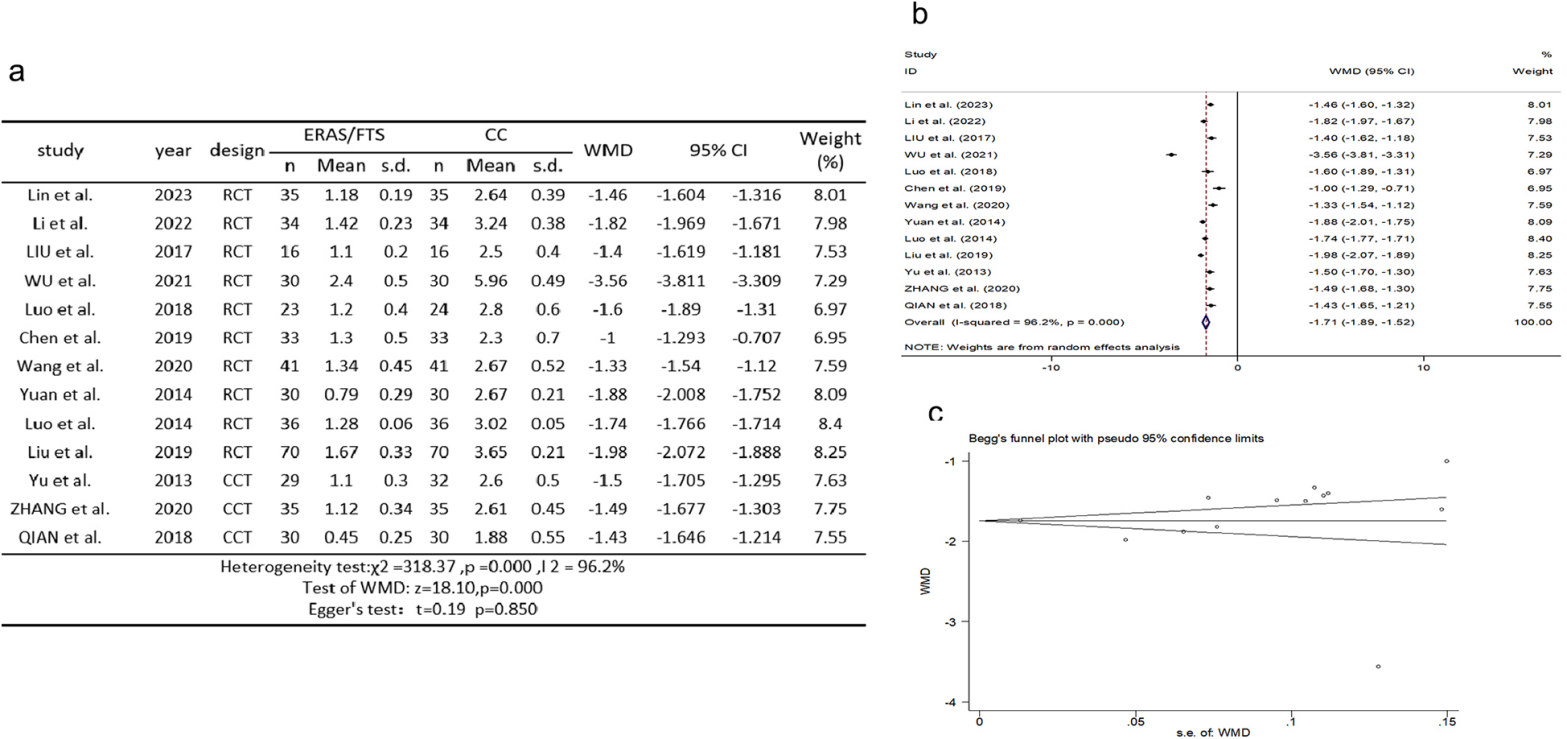
Association between ERAS or FTS and time of first enteral nutrition after operation in the perioperative period of acute gastrointestinal perforation. **(a)** Results of the meta-analysis of the association between ERAS or FTS and time of first enteral nutrition after operation; **(b)** odds ratio in positive for ERAS or FTS; **(c)** Egger's funnel plot of studies investigating ERAS or FTS as a risk factor.

Thirteen studies (2, 10, 11, 14, 15, 17, 18, 22–24, 29–31) reported a comparison of first postoperative enteral nutrition times. There was heterogeneity among the studies (*p* = 0.000, *I*^2^ = 96.2%). A random-effects model was used for meta-analysis. The results showed that the time to first postoperative enteral nutrition was advanced in the ERAS group compared with the control group (WMD = −1.709, 95% CI: −1.894 to −1.524, *p* = 0.000); Egger's test showed no publication bias (*t* = 0.19, *p* = 0.850), as shown in Figure 4.

### Time of first out-of-bed activity after operation

**Figure 5:**
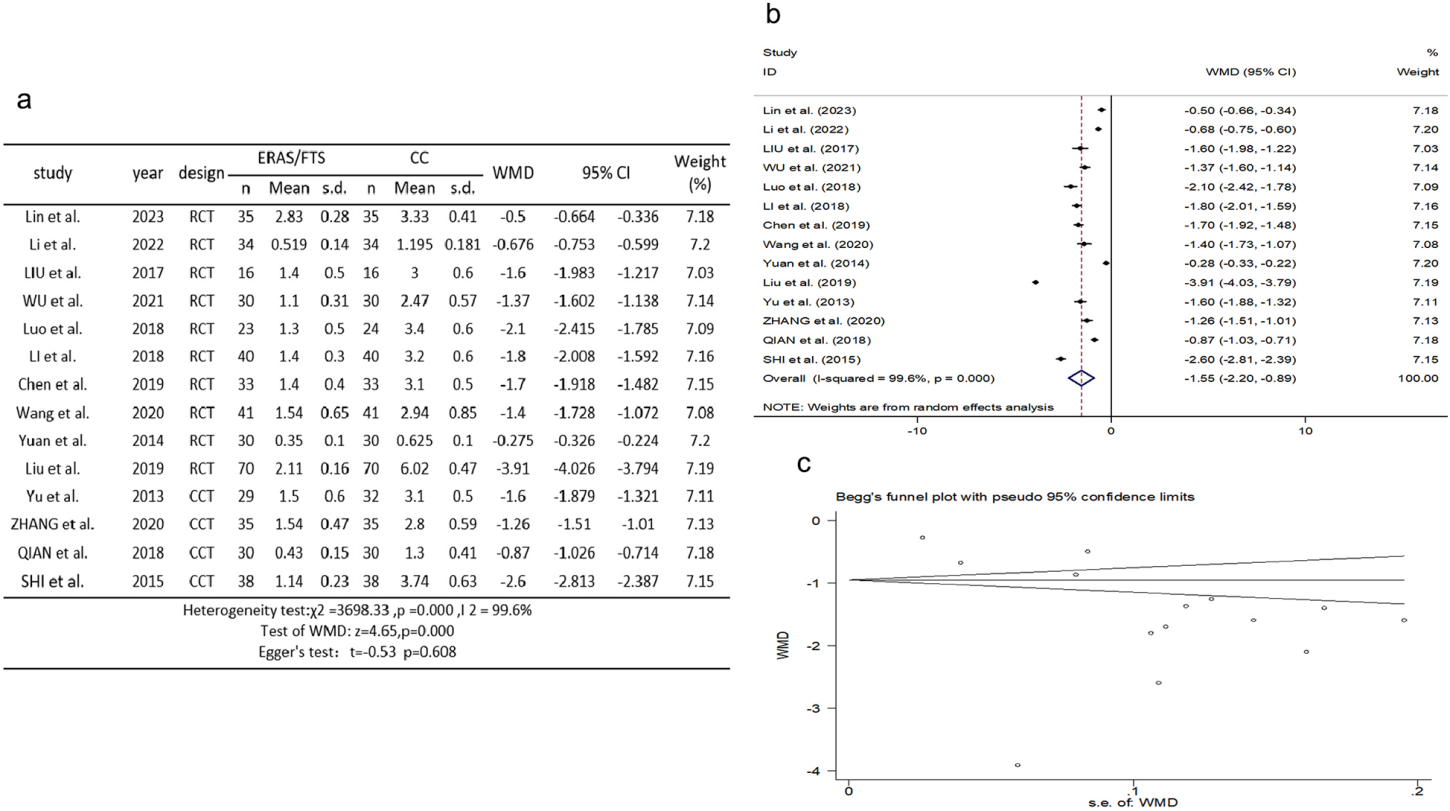
Association between ERAS or FTS and time of first out-of-bed activity after operation in the perioperative period of acute gastrointestinal perforation. **(a)** Results of the meta-analysis of the association between ERAS or FTS and time of first out-of-bed activity after operation; **(b)** odds ratio in positive for ERAS or FTS; **(c)** Egger's funnel plot of studies investigating ERAS or FTS as a risk factor.

Fourteen studies (2, 10, 11, 14–18, 22, 24, 29–31, 34) reported a comparison of the time of first postoperative ambulation. There was heterogeneity among the studies (*p* = 0.000, *I*^2^ = 99.6%). A random-effects model was used for meta-analysis. The results showed that the ERAS group had an earlier time of first postoperative out-of-bed activity compared with the control group (WMD = −1.546, 95% CI: −2.198 to −0.895, *p* = 0.000); Egger's test showed no publication bias (*t* = −0.53, *p* = 0.608), as shown in Figure 5.

### Incidence of postoperative complications

**Figure 6:**
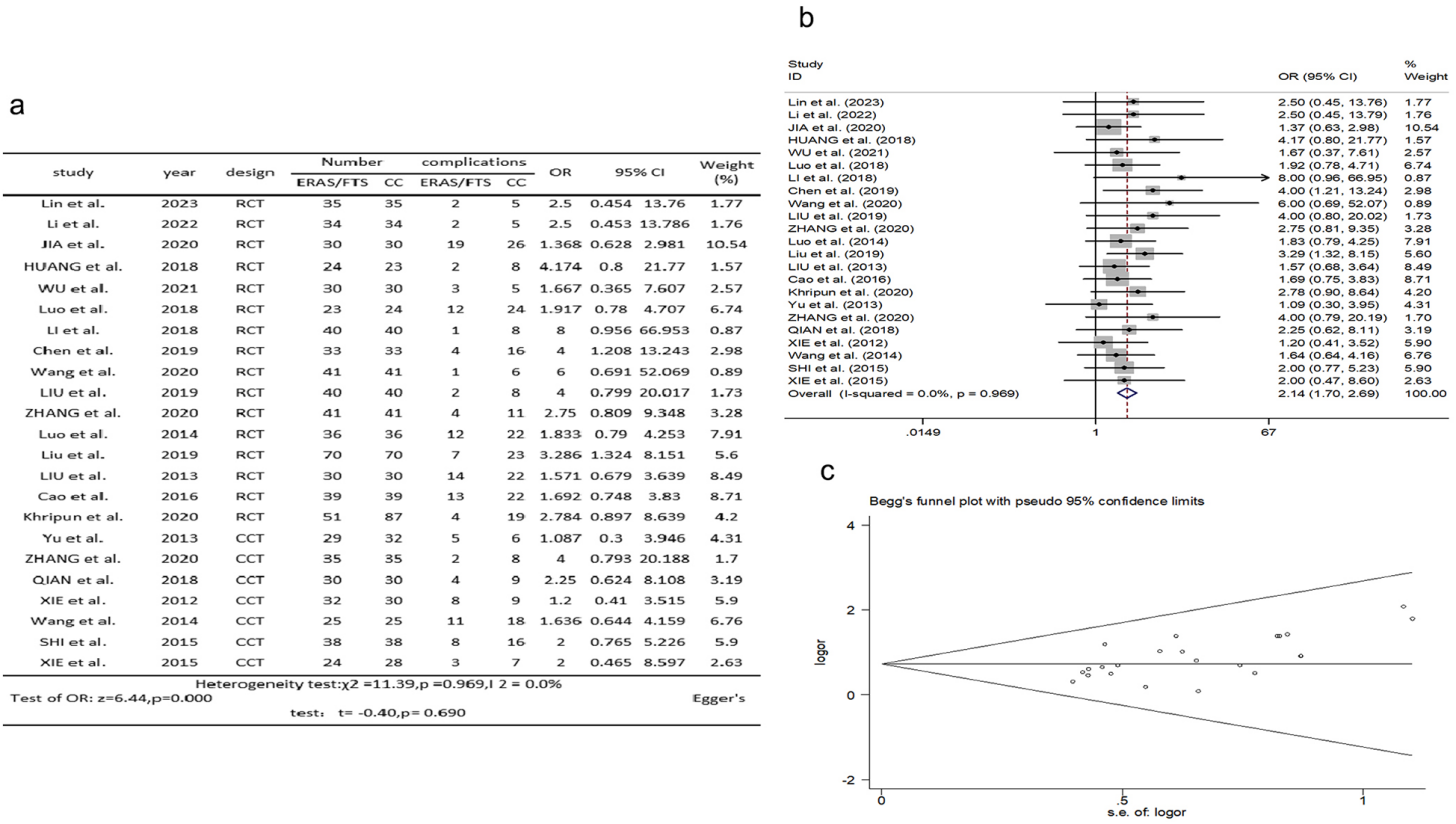
Association between ERAS or FTS and incidence of postoperative complications in the perioperative period of acute gastrointestinal perforation. **(a)** Results of the meta-analysis of the association between ERAS or FTS and Incidence of postoperative complications; **(b)** odds ratio in positive for ERAS or FTS; **(c)** Egger's funnel plot of studies investigating ERAS or FTS as a risk factor.

Twenty-three studies (2, 10, 12–20, 23–24, 26–35) reported comparisons of postoperative complication rates. There was no significant heterogeneity among the studies (*p* = 0.969, *I*^2^ = 0.0%). A fixed-effects model was used for meta-analysis. The results showed a lower rate of postoperative complications in the ERAS group compared with the control group (OR = 2.137, 95% CI: 1.696–2.693, *p* = 0.000); Egger's test showed no publication bias (*t* = −0.40, *p* = 0.690), as shown in Figure 6.

### Hospitalization time

**Figure 7:**
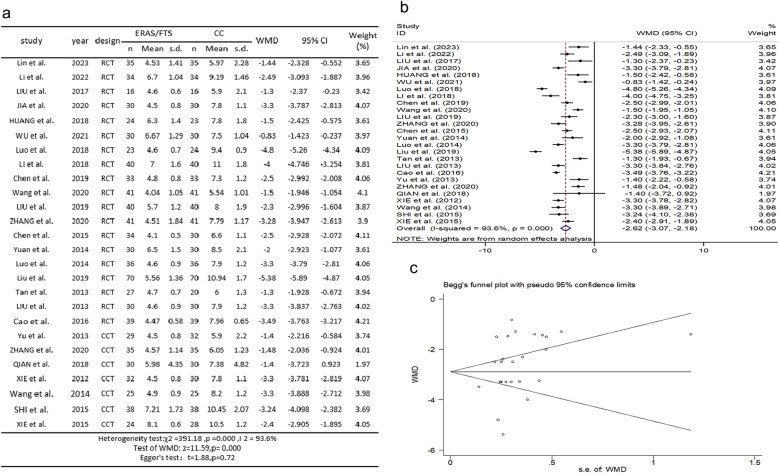
Association between ERAS or FTS and hospitalization time in the perioperative period of acute gastrointestinal perforation. **(a)** Results of the meta-analysis of the association between ERAS or FTS and Hospitalization time; **(b)** odds ratio in positive for ERAS or FTS; **(c)** Egger's funnel plot of studies investigating ERAS or FTS as a risk factor.

Twenty-six studies (2, 10–27, 29–35) reported comparisons of length of stay. There was heterogeneity among the studies (*p* = 0.000, *I*^2^ = 93.6%). A random-effects model was used for meta-analysis. The results showed that the length of hospital stay was shorter in the ERAS group compared with the control group (WMD = −2.624, 95% CI: −3.068 to −2.181, *p* = 0.000); Egger's test showed no publication bias (*t* = 1.88, *p* = 0.72), as shown in Figure 7.

### Hospitalization expenses

**Figure 8:**
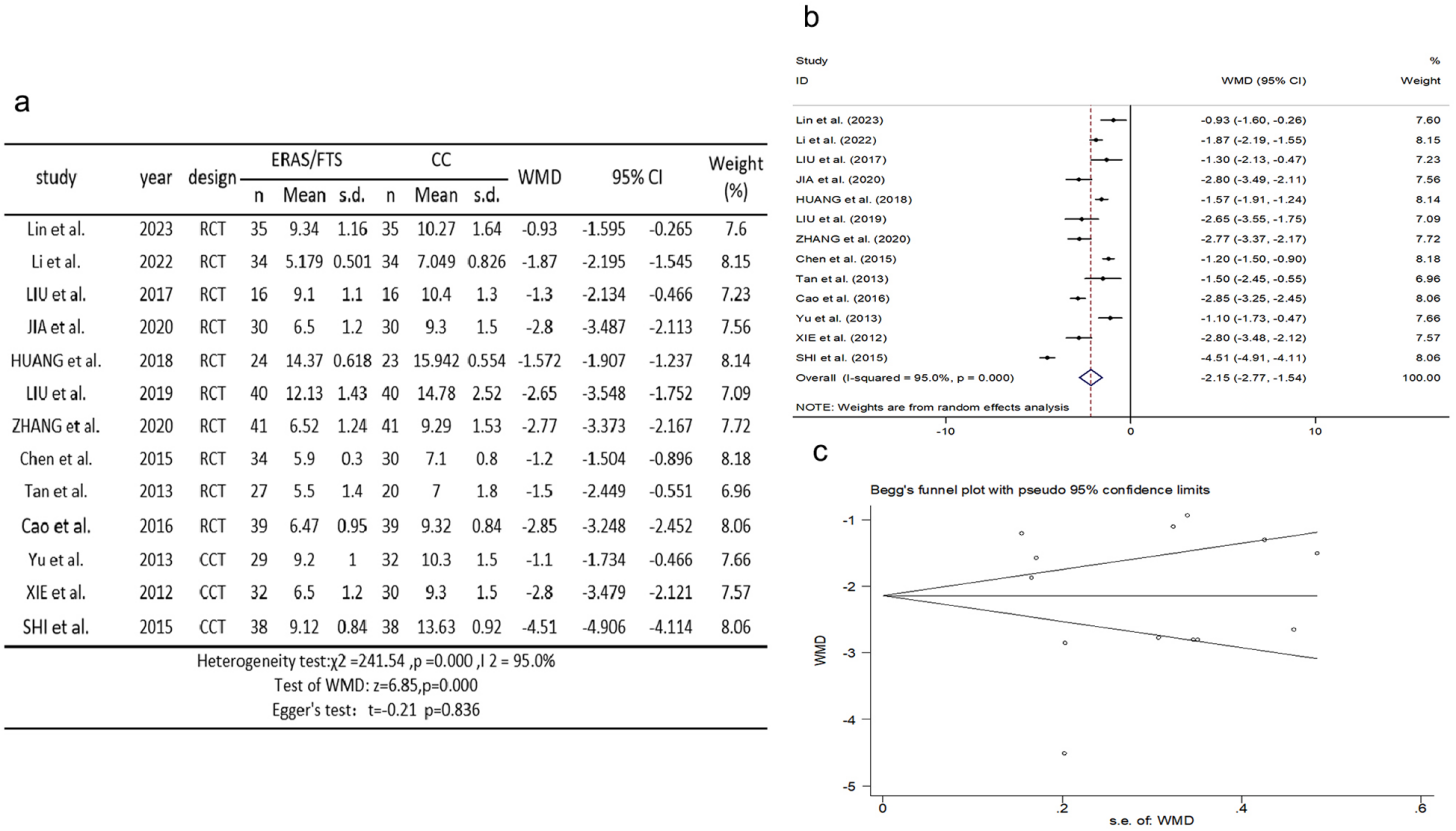
Association between ERAS or FTS and hospitalization expenses in the perioperative period of acute gastrointestinal perforation. **(a)** Results of the meta-analysis of the association between ERAS or FTS and Hospitalization expenses; **(b)** odds ratio in positive for ERAS or FTS; **(c)** Egger's funnel plot of studies investigating ERAS or FTS as a risk factor.

Thirteen studies (2, 10–13, 19–21, 25, 27, 29, 32, 34) reported comparisons of hospital costs. There was heterogeneity among the studies (*p* = 0.000, *I*^2^ = 95.0%). A random-effects model was used for meta-analysis. The results showed that the ERAS group had less hospital costs compared with the control group (WMD = −2.151, 95% CI: −2.767 to −1.535, *p* = 0.000); Egger's test showed no publication bias (*t* = −0.21, *p* = 0.836), as shown in Figure 8.

## Discussion

The core idea of ERAS is to reduce the stress reaction, maintain the homeostasis of the body, reduce the incidence of surgical complications and mortality, and promote the recovery of patients. The implementation of this concept requires a multidisciplinary team (MDT) of surgeons, anesthesiologists, physiotherapists, nurses, etc. (36). The main contents include (37) (1) preoperative management, including preoperative education, nutritional screening, prophylactic use of antibiotics, prevention of stress gastric mucosal lesions and antithrombotic therapy, individualized control of blood pressure and blood glucose, and corresponding management programs; (2) intraoperative management, including minimally invasive surgery, optimal anesthesia, limited fluid replacement, thermal preservation during operation, blood glucose control, prevention of postoperative nausea and vomiting, thrombosis of lower limbs, and stress-induced mucosal lesions; and (3) postoperative management, including postoperative monitoring, catheter management, incision management, promoting intestinal function recovery and early activity, and nutritional support.

The results of the meta-analysis showed that the application of the ERAS/FTS concept in the perioperative period of acute digestive tract perforation could significantly reduce the incidence of stress reaction, pain reaction, and complications compared with the traditional treatment group; the time of getting out of bed for the first time, the time of the first exhaust, and the time of taking food after operation were earlier than those in the traditional treatment group. It shortens the hospitalization time of patients, accelerates the postoperative rehabilitation of patients, improves the effective utilization rate of hospital beds, and reduces the cost of hospitalization and the economic burden. Sensitivity analysis was performed on the age of patients, and after excluding different studies in turn, there was no significant difference between the results of the meta-analysis of the remaining studies and those before excluding, showing that age had no significant effect on the results. However, the ERAS/FTS does not significantly reduce operative time and intraoperative blood loss, which requires further improvement in the implementation of the ERAS/FTS concept. We will continue to pay attention to and further efforts to search for more reports of these outcomes.

All the studies included in the meta-analysis met the inclusion and exclusion criteria, and the ERAS groups of each study were comparable with the control group, but the following limitations still exist: (1) due to the limited sample size in RCTs, this study included seven CCT studies as case–control studies. This inclusion may lead to either overestimation or underestimation of the outcomes. Additionally, two RCTs and two CCTs did not perform allocation concealment, which potentially caused selection bias and lowered the quality of the literature. (2) The literature included in the studies used different ERAS measures. There is no uniform standard for the specific implementation method; there is a greater subjectivity, which may have a greater impact on the homogeneity of the study; and there may be implementation bias. (3) There were differences in the degree of ERAS protocol implementation and surgical technique proficiency among different research centers. (4) There are individual differences in the condition of patients themselves. (5) The blind method of some included studies was unclear and may have measurement bias. (6) Funnel plot analysis has some publication bias. Funnel plot analysis was performed for the incidence of complications, the distribution on both sides of the funnel was basically symmetrical, and the points were distributed within the inverted funnel, indicating that the impact of publication bias on the results was small.

In conclusion, the application of the ERAS/FTS concept in the perioperative period of acute digestive tract perforation can reduce postoperative complications, promote the recovery of patients, and shorten the length of hospital stay and hospital costs. This approach offers a certain degree of safety and effectiveness while saving medical resources and reducing the societal and familial burden. Widespread promotion and application of ERAS/FTS in emergency surgery could yield significant economic benefits and greatly benefit emergency patients. The prospects for application are broad and worth promoting. Because ERAS protocols, surgical techniques, and sample populations vary between individuals, large-scale multicenter RCTs with standardized ERAS protocols are necessary to provide further evidence for clinical practice guidelines. This study lacks standardized ERAS protocols, as the therapeutic measures adopted in each study were not uniform, leading to varying degrees of ERAS implementation. This limitation introduces a risk of bias that may affect the accuracy of the results. More clinical samples and multicenter, high-quality RCTs are needed to further evaluate and provide evidence for clinical practice guidelines. Enhanced recovery after surgery requires multidisciplinary collaboration and depends heavily on close cooperation and good organization of patients, their families, medical staff, anesthesia, nursing, operating room, intensive care unit, and other departments. ERAS is essentially an MDT approach in the field of surgery, emphasizing the collaboration and integration of various disciplines, including surgery, anesthesia, nursing, and other disciplines.

## Data Availability

The original contributions presented in the study are included in the article/Supplementary Material, further inquiries can be directed to the corresponding authors.
